# Linarin, a Glycosylated Flavonoid, with Potential Therapeutic Attributes: A Comprehensive Review

**DOI:** 10.3390/ph14111104

**Published:** 2021-10-29

**Authors:** Javad Mottaghipisheh, Hadi Taghrir, Anahita Boveiri Dehsheikh, Kamiar Zomorodian, Cambyz Irajie, Mohammad Mahmoodi Sourestani, Aida Iraji

**Affiliations:** 1 Center for Molecular Biosciences (CMBI), Institute of Pharmacy/Pharmacognosy, University of Innsbruck, Innrain 80-82, 6020 Innsbruck, Austria; 2 Department of Medicinal Chemistry, Faculty of Pharmacy, Shiraz University of Medical Sciences, Shiraz 71468-64685, Iran; hadi_taghrir@yahoo.com; 3 Department of Horticultural Science, Faculty of Agriculture, Shahid Chamran University of Ahvaz, Ahvaz 61357-43311, Iran; anahitaboveiri84@gmail.com (A.B.D.); m.mahmoodi@scu.ac.ir (M.M.S.); 4 Department of Medical Mycology and Parasitology, School of Medicine, Shiraz University of Medical Sciences, Shiraz 14336-71348, Iran; kzomorodian@gmail.com; 5 Department of Medical Biotechnology, School of Advanced Medical Sciences and Technologies, Shiraz University of Medical Sciences, Shiraz 71348-14336, Iran; irajie@sums.ac.ir; 6 Central Research laboratory, Shiraz University of Medical Sciences, Shiraz 71348-14336, Iran; 7 Stem Cells Technology Research Center, Shiraz University of Medical Sciences, Shiraz 71348-14336, Iran

**Keywords:** flavonoids, linarin, chemotaxonomy, phytochemistry, bioactivities

## Abstract

Many flavonoids, as eminent phenolic compounds, have been commercialized and consumed as dietary supplements due to their incredible human health benefits. In the present study, a bioactive flavone glycoside linarin (LN) was designated to comprehensively overview its phytochemical and biological properties. LN has been characterized abundantly in the *Cirsium*, *Micromeria*, and *Buddleja* species belonging to Asteraceae, Lamiaceae, and Scrophulariaceae families, respectively. Biological assessments exhibited promising activities of LN, particularly, the remedial effects on central nervous system (CNS) disorders, whereas the remarkable sleep enhancing and sedative effects as well as AChE (acetylcholinesterase) inhibitory activity were highlighted. Of note, LN has indicated promising anti osteoblast proliferation and differentiation, thus a bone formation effect. Further biological and pharmacological assessments of LN and its optimized semi-synthetic derivatives, specifically its therapeutic characteristics on osteoarthritis and osteoporosis, might lead to uncovering potential drug candidates.

## 1. Introduction

The application of plants for medicinal purposes is as old as humanity itself. Since many of them are considered as functional foods and extensively consumed in folk medicine, their biological and phytochemical assessments are pivotal attitudes [1,2]. By developing human knowledge, the study of plant constituents has led to the discovery of secondary metabolites (phytochemicals) as the major compounds responsible for the bioactivities. These biosynthesized compounds (both volatile and non-volatile) mostly possess defensive roles in plants to assist surviving them against abiotic and biotic stressors [3,4].

Investigation of phytoconstituents has been the target of many researchers in order to determine their health benefits. So far, many phytochemicals have been developed and consumed as successful drugs for the treatment of a diverse range of ailments and disorders, specifically cancer types [5,6,7]. Among the varied phytochemical classifications, flavonoids have been introduced as one of the largest natural phenolic compounds with broad valuable biological properties [8,9]. Based on the chemical structures, these compounds are divided into six main subclasses: flavones, flavanones, flavonols, flavan-3-ols, isoflavones, and anthocyanins [10,11]. Even though the phytochemical and biological characteristics of these compounds are being studied [11,12], they are still interesting target molecules to be explored.

**Figure 1:**
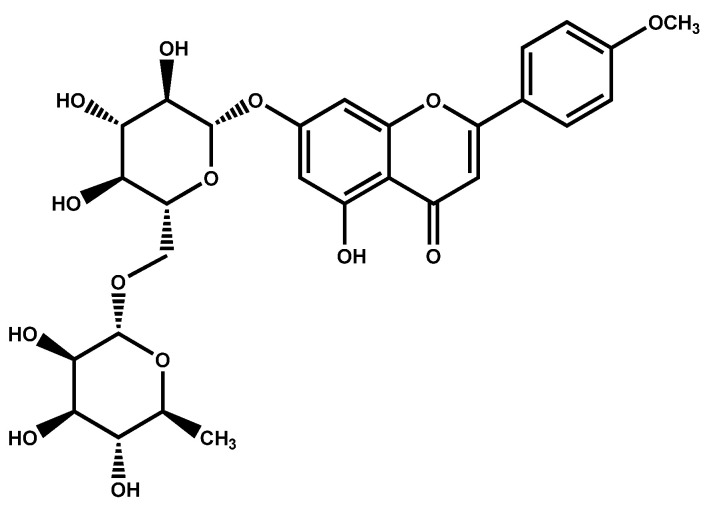
The chemical structure of linarin.

Linarin (syn. acacetin 7-O-rhamnosyl(1’′′→6′′)glucoside, or acacetin 7-O-rutinoside), as a glycosylated flavone (Figure 1), has been identified from various plant species mainly belonging to the Asteraceae and Lamiaceae families. Regarding the potent bioactivities of this flavonoid reported by several experiments, and the importance of flavonoid consumption as drugs and/or supplements, the present study aims at comprehensively collecting all the phytochemical (i.e., chemotaxonomy and phytochemistry) and biological reports of this flavonoid.

The scientific databases including Web of Science, SciFinder, and PubMed were used to find the correlated data by utilizing the keyword of “linarin” within the English-language papers (access date: 25 May 2021).

## 2. Phytochemistry and Chemotaxonomy of Linarin

So far, among the 13 plant families containing linarin (LN), Asteraceae and Lamiaceae have been identified as the richest ones. The most LN contents have been reported in various *Cirsium* spp.; however, this compound has also been isolated from the genus *Micromeria* and *Buddleja* belonging to Lamiaceae and Scrophulariaceae, respectively. This glycosylated flavone has mainly been isolated and characterized from alcoholic (methanolic and ethanolic) and hydro-alcoholic extracts. In the following sections, the available data on the phytochemistry of this compound are discussed in detail (Tables S1 and S2).

### 2.1. Isolation of Linarin from Plant Species

#### 2.1.1. Asteraceae

LN has been isolated from diverse parts of *Cirsium* spp. By utilizing column chromatography on silica gel (CC) as the final separation step, this compound has been isolated from the methanolic extract of *C. arvense* aerial parts [13]. From the roots of *C. arvense* subsp. *vestitum* via application of vacuum column chromatography [14], and flowers of *C. canum* (L.) using reverse-phase high-performance liquid chromatography (RP-HPLC), LN has been isolated [15].

*C. japonicum* can be considered as one of the richest plant species of LN. Sephadex^®^ LH-20 (SLH) has been applied to the isolation or purification of many flavonoid derivatives [12]. This technique has been employed to isolate LN from the aerial parts of *C. japonicum* [16].

Zhang et al. (2018) isolated LN from *C. japonicum* [17]; in addition, liquid chromatography-mass spectrometry (LC-MS/MS) was implemented to characterize it from the hydro-ethanolic (70%) extract [18]. Preparative-HPLC has been exploited to isolate LN from the ethanolic fraction of *C. japonicum* var. *maackii* [19]. Moreover, the methanolic extract of *C. japonicum* var. *ussuriense* (Regel) Kitam. ex Ohwi obtained from the aerial parts has been subjected to isolate LN by applying the solvent system of CHCl_3_−MeOH–H_2_O (25:8:5) in CC on silica gel [20].

LN has been isolated from three other *Cirsium* species: from the leaf and flower methanolic extract of *C. rivulare* using preparative-HPLC [21]; from the flower methanolic fraction of *C. setidens* applying CC on Silica gel [22]; and from the aerial arts using liquid chromatography (LC) [23] and hydro-ethanolic extracts of *C. setosum* (Willd.) MB. (utilizing HPLC) [24]; however, LN has also been identified from the ethanolic extract of this species by applying ultra-performance liquid chromatography-mass spectrometry (UPLC–MS) [25].

*Chrysanthemum* species are considered as one of the major sources of LN. It has been isolated from the methanolic extracts of *Chrysanthemum* *boreale* (Makino) Makino flowers by utilizing CC on silica [26,27], and the hydro-ethanolic (95%) fractions obtained from the *Chrysanthemum morifolium* Ramat flowers [28].

*C. indicum*, famed as “Ye Ju Hua” in China, has a long history in the treatment of inflammation, hypertension, and respiratory diseases in traditional Chinese and Korean medicine; furthermore, it is traditionally used in tea preparations, tinctures, creams, and lotions [29].

This plant species (*C. indicum*) has been implemented to isolate LN conducted by several studies. It has also been isolated from its flower, using mostly CC on silica gel [30,31,32,33], from the dichloromethane extracts of aerial part and methanolic soluble-fraction of the whole part via application of CC on silica gel [34,35].

The purification of LN was also carried out by the solid-liquid extraction method from the hydroethanolic (75%) extract of the same plant species through utilization of various solvents including petroleum ether, ethyl acetate, ethanol, and water [36]. The whole herb and its aerial parts of *C. zawadskii* var. *latilobum* (Maxim.) Kitam. has been reported to possess LN, whereas CC on silica gel was used [37,38].

In the study of Li et al. (2016), high-speed counter-current chromatography (HSCCC) was applied in order to isolate this flavonoid from the hydro-ethanolic extract (80%) of *Flos Chrysanthemi indici* [39], however, it has also been identified from this species as reported by three other groups [40,41,42].

The whole part methanolic extract of *Artemisia capillaris* Thunb. has been chromatographed by CC on silica gel using CH_2_Cl_2_–MeOH (20:1) as solvent systems leading to isolate LN [43]; moreover, this compound was identified in a rare species *Picnomon acarna* (L.) Cass., where its aerial parts were separated in CC [44].

#### 2.1.2. Lamiaceae

Lamiaceae (syn. Labiatae), a large plant family consisting of perennial or annual herbaceous plants and shrubs, is majorly known for their aromatic characteristics [45]. Various genus belonging to this family are considered as natural flavonoid sources including LN. Among them, different species of *Mentha*, *Micromeria*, and *Satureja* can be mentioned.

LN has previously been isolated from the hydro-methanolic (80%) extracts of the flower [46] and aerial parts [47] of *Mentha arvensis* L.; however, it has been reported in *M. haplocalyx* Briq. in the ethyl acetate extracts of the aerial parts of three other *Mentha* species comprising *M. spicata*, *M. piperita*, and *M. villosonervata*, where CC on silica gel was applied as the final chromatography step [48].

Dai et al. (2008) isolated LN from a hydro-ethanolic (75%) soluble-fraction of *Dracocephalum peregrinum* L. aerial parts by hiring extensive chromatographic techniques [49]. This flavone has previously been isolated and characterized from other following species: ethanolic extract of *Leonurus japonicus* Houtt. aerial parts (via CC on silica gel CH_2_Cl_2_–MeOH (100:1–0:100) [50] as well as the leaves of methanolic extracts of *Calamintha officinalis* Moench [51] and *Calamintha glandulosa* (Req.) Benth. [52], where in the later study, semiprep-HPLC was utilized as the final separation step by using H_2_O–ACN (50 to 100%). LN has further been reported in the *Ziziphora clinopodioides* Lam. herb methanolic extract [53].

#### 2.1.3. Scrophulariaceae

The plants belonging to Scrophulariaceae can be considered as the third natural source of LN. Among them *Buddleja* spp. are the richest ones. Previously, from the leaf methanolic extract of *Buddleja davidii* Franch., LN was isolated by the utilization of centrifugal partition chromatography (CPC) and the solvent system of CHCl_3_–MeOH–H_2_O (45:33:22) [54]. El-Domiaty et al. (2009) also purified LN from the whole part hydro-ethanolic (95%) extract of *Buddleja asiatica* Lour., while CC on silica gel was used to separate it [55].

*Buddleja cordata* Kunth was subjected to isolate its phytoconstituents and LN was isolated and characterized from the leaves [56] and whole parts [57]. In three other investigations, LN was isolated from mostly alcoholic extracts of the flowers of *Buddleja officinalis* Maxim [58,59,60]. CC on silica gel using CHCl_3_–MeOH with ratios of 19:1, 9:1, 8:2 were applied to isolate LN from the aerial parts of *Buddleja scordioides* Kunth [61]. This compound was isolated from two *Linaria* species *L. japonica*, *L. vulgaris*, and *L. kurdica* subsp. *eriocaly*, while the whole parts were chromatographed [62,63,64].

#### 2.1.4. Miscellaneous Plants

LN has been isolated from the whole part methanolic extract of *Exacum macranthum* Arn. ex Griseb. (Gentianaceae) via the recrystallization method [65]. This phytochemical has also been isolated and identified from *Lobelia chinensis* Lour. (Campanulaceae) [66], *Ginkgo biloba* L. (Ginkgoaceae) [67], *Bombax malabaricum* DC. (Malvaceae) [68], *Avena sativa* L.(Poaceae) [69], *Thalictrum aquilegiifolium* L. [70], and *Coptis chinensis* Franch [71] (Ranunculaceae), *Zanthoxylum affine* Kunth (Rutaceae) [72] and *Lippia rubella* (Moldenke) T.R.S.Silva & Salimena (Verbenaceae) [73].

### 2.2. Quantification and Qualification Analysis of Linarin in Plants

By utilization of extensive analytical methods, LN has been qualified and quantified in plant species. So far, the plants belonging to Asteraceae, Lamiaceae, Scrophulariaceae, and Valerianaceae have been reported to be rich in LN content. Table S2 comprehensively lists all the information regarding the fingerprinting analysis of this compound throughout the plant species, however, the following sections describe them in brief.

#### 2.2.1. Asteraceae

Plants in the Asteraceae family, particularly *Cirsium* spp. and *Chrysanthemum* spp., have been characterized as the richest herbal sources of LN. It has been identified throughout six species of the *Cirsium* genus; HPLC coupled to an ultraviolet (UV) detector was used to qualify this compound in the methanolic extract of *C. arvense* [13], along with the report by Demirta et al. (2017), which quantified LN from its root via HPLC-MS (MicroTOF-Q) [14].

The LN content of various soluble-fraction extracted from the flower part of *Cirsium canum* (L.) All. has formerly been analyzed by HPLC-DAD (HPLC-diode array detector). Consequently, the hydro-methanolic (50%) and dichloromethane extracts possessed the highest and lowest contents with 121.75 and 1.94 µg/g, respectively [15].

*Cirsiumjaponicum* (Thunb.) Fisch. ex DC., Japanese field thistle, is renowned in Chinese pharmacopeia for the treatment of inflammation and bleeding [16] as well as application in Korean folk medicine as a uretic as well as antihemorrhagic and antihepatitic medication [74]. Nonetheless, Ganzera et al. (2005) analyzed pectolinarin as the main phytoconstituent of the *C. japonicum* methanolic aerial part extract, and LN was also quantified with a significant content of 0.26–1.15 mg/100 g through different plant samples by employing HPLC-MS [16].

From the alcoholic extracts of two different *C. japonicum* varieties (*C. japonicum* var. *maackii* Maxim and *C. japonicum* var. *ussuriense* (Regel) Kitam. ex Ohwi), LN was detected by employing HPLC-UV [19,20]. Moreover, the mixture of LN and pectolinarin was compared with the methanolic extracts obtained from the leaf (170 mg/g) and flower (20 mg/g) parts of *Cirsium rivulare* (Jacq.) All., whereas HPLC-UV was utilized as the analytical tool [21]. The methanolic extract of *Cirsiumsetidens*(Dunn) Nakai was phytochemically analyzed through HPLC-UV and a significant LN concentration of 120.3 mg/g was measured [22].

*Cirsiumsetosum* (Willd.) Besser ex M.Bieb. has further been elaborated to possess phytochemical contents in four studies. The LN content range of 0.3–2 mg/100 g has been recorded through analysis with HPLC-MS [16]. The methanolic soluble partitions of *Hemistepta lyrate* (Bunge) Bunge flower extracted from different plant samples were analytically assessed, and LN was subsequently quantified (0.06–4.26 mg/g) [26].

In a comparative phytochemical analysis of the ethanolic extract obtained from the *Chrysanthemum* *morifolium* Ramat. flower, LN was qualified and quantified in three cultivars by using HPLC-DAD-ESI/MS with the contents ranging from 0.117 to 0.583 mg/g [28]. HPLC-DAD analysis of the *Chrysanthemum zawadskii* var. *latilobum* (Maxim.) Kitam. extract showed LN as the marker compound with a 22.8 mg/g extract [75].

*Chrysanthemum indicum* L., as an edible medicinal plant, is famed for its consumption as a food supplement and herbal tea. It has a diverse range of therapeutic applications, specifically in Chinese and Korean folk medicine, for the treatment of immune-related disorders, to heal several infectious diseases, and hypertension symptoms [31]. In several studies reporting its phytoconstituents, LN has also been characterized as the major compounds. He et al. (2016) qualified this compound in the flower methanolic extract via utilization of HPLC-DAD [31].

In a comparative investigation, the phytochemical content of different parts of *C. ndicum* dichloromethane extract was analyzed. As the result, the leaf extract contained the highest LN content (1.47 g/100 g) compared to its stem and flower parts (0.65 g/100 g) [35]. In a similar study, HPLC-MS application led to the fingerprinting analysis of various *C. indicum* parts collected from China; consequently, the root and flower parts indicated the highest and lowest LN amounts (0.344 and 0.052 μg/mg FW), respectively [34]. Furthermore, the hydro-ethanolic extract (75%) of several *C. indicum* samples was phytochemically assessed by HPLC-DAD, and a diverse range of LN concentrations (2.08–55.68%) was recorded [36]. Apart from a qualification study, in which the LN content was determined in the flower methanolic extract of *C. indicum* [32], hydro-ethanolic partition (95%) of the flower and bud parts contained 48.3 mg/g, whilst acetonitrile and water (in formic acid 0.1%) in HPLC-DAD was used as the solvent system [30].

The flower hydro-ethanolic extract (80%) was analyzed via HPLC-UV and LN was accordingly quantified (32.8 mg/g) [39]. The impacts of several extraction conditions on LN contents of the *C. indicum* flower ethanolic extract [40] have been explored; the highest LN yield (88.11%) was measured in the plant samples extracted with 80% ethanol, 2 h of extraction, extraction frequency of three, and solvent to material ratio of 12 mL/g [40].

HPLC-DAD-MS was formerly employed to analyze LN in the hydro-methanolic (60%) extract of *C. indicum* [41]; in addition, a method for fingerprinting analysis of its methanolic extract via HPLC-DAD was introduced by Jung et al. (2012), where it contained 14.6–15.3 µg/g [42].

#### 2.2.2. Lamiaceae

Lamiaceae, as the second richest LN natural source, has been analytically investigated by diverse groups. The occurrence of this flavone glycoside has been confirmed in various *Mentha* species. The flower methanolic fraction of *M. arvensis* was formerly analyzed and 6% of LN content was reported [46]. In a quantification measurement, the hydro-methanolic (80%) extract of aerial parts of *M. arvensis* was subjected to HPLC-DAD and UPLC-ESI/Q-TOF/MS, and the LN presence was validated [47].

This compound was further detected in the *Menthahaplocalyx* (Briq.) Trautm. extract (via HPLC-MS/MS) [76]; furthermore, Erenler et al. (2018) comparatively analyzed the ethyl acetate aerial part extracts of three other *Mentha* species including *M. haplocalyx*, *Menthaspicata* L., and *Mentra* x *piperita* L. The highest and lowest LN contents were observed in *M. spicata* and *M. piperita* samples with 42.21 and 0.04 mg/g, respectively [48].

Marin et al. (2001), by using HPLC-UV, detected LN in the leaf hydro-methanolic (80%) extracts of the following plant species: *Acinos arvensis* ssp. *villosus*, *Acinos. Hungaricus* (Simonk.) Šilic, *Calamintha glandulosa* (Req.) Benth., *Micromeria albanica* (K.Malý) Šilić, *Micromeria cristata* (Hampe) Griseb., *Micromeria dalmatica* Benth., *Micromeria juliana* (L.) Benth. ex Rchb., *Micromeria thymifolia* (Scop.) Fritsch, *Satureja cuneifolia* Ten., *Satureja kitaibelii* Wierzb. ex Heuff., and *Satureja montana* ssp. *montana*. Moreover, the leaf hydro-methanolic fraction of *Calamintha officinalis* Moench was phytochemically analyzed through HPLC-UV and water–acetonitrile, and methanol as solvents, and LN with a concentration of 0.27 mg/g was identified [51].

The chemical composition of *Ziziphora clinopodioides* Lam. has further been studied analytically; UPLC-Q-TOF-MS was utilized to detect LN from its hydro-ethanolic (70%) extract [77] as well as the quantification analysis of the herb methanolic fraction, where by applying RP-RRLC (RP-rapid resolution liquid chromatography), the LN contents were detected (3.15–20.55 mg/g) [53].

#### 2.2.3. Scrophulariaceae

*Buddleja* spp. has been analytically elaborated in the case of its phytoconstituents; consequently, LN was detected as one of the main compounds. Fan et al. (2008) identified LN in the leaf methanolic fractions of *Buddleja davidii* Franch. and *Buddleja nitida* Benth., where LC-MS/MS was used. LN concentration of the ethanolic extracts (70%) was assessed in the leaf and in vitro culture samples of *Buddleja* *cordata* Kunth including white and green callus and root samples by using HPLC-DAD, and the highest content was detected in the leaf ethanolic extract (41.81 ± 5.21 mg/g) [56]. The hydro-ethanolic (70%) fraction of *Buddleja officinalis* Maxim. flower was analyzed via utilization of UHPLC-LTQ-Orbitrap, and LN was consequently qualified [59]. The lyophilized infusion prepared from *Linaria vulgaris* Mill. has previously been experimented through HPLC-UV and LN was quantified with a significant content of 3.84 g/kg drug [63].

#### 2.2.4. Valerianaceae

*Valeriana* spp. has been characterized for its LN content. In [78], by applying HPLC-DAD, they analytically investigated six *Valeriana* species (*Valeriana edulis* Nutt., *Valeriana officinalis* L., *Valeriana jatamansi* Jones, *Valeriana procera* Kunth, and *Valeriana sitchensis* Bong.), with the highest and lowest LN content detected in the methanolic extracts of *V. jatamansi* and *V. edulis* with 0.24 and <0.002%, respectively.

#### 2.2.5. Miscellaneous Plants

The methanolic extracts of the *Lobelia chinensis* Lour. herb belonging to the Campanulaceae family were characterized for its phytochemicals by two analytical tools (LC-MS and HPLC-DAD-MS), and LN was qualified [66]. The hydro-ethanolic (70%) fractions extracted from the inflorescence part of *Coptis chinensis* Franch. (Ranunculaceae family) were assessed via HPLC-MS and LN was identified as the main compounds [71]. Moreover, Rios et al. (2018) identified LN in the hexane, acetone, and methanolic extracts of *Zanthoxylum affine* Kunth (Rutaceae) aerial parts, whilst HPLC−Q-TOF-MS was employed with water and methanol as the solvent systems [72].

## 3. Biological Properties of LN

**Figure 2:**
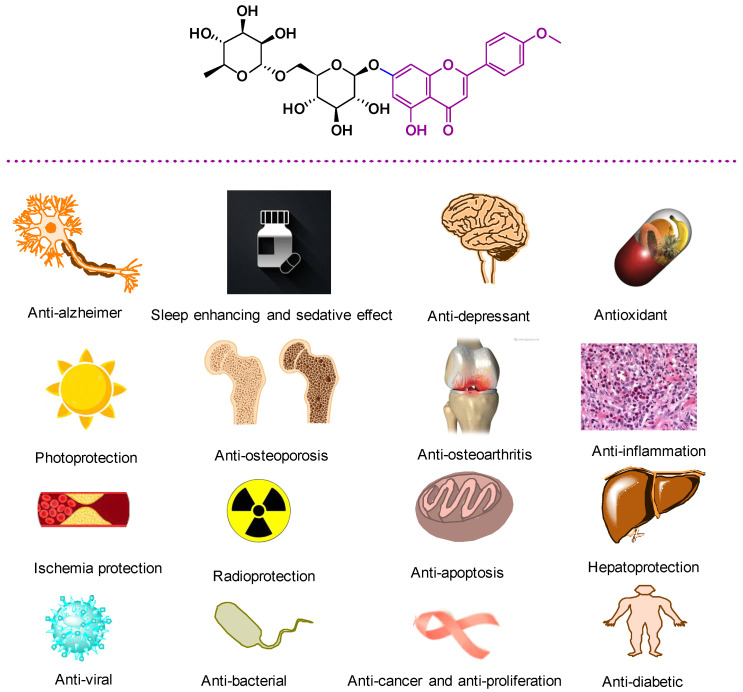
Summary of the biological activities of LN.

Generally, LN is still a relatively un-investigated drug resource. As a result, in this section, the therapeutic potential of LN and LN containing plants is summarized in Figure 2 and are classified according to which could be useful for potential clinical applications (Table S3).

### 3.1. Anti-Alzheimer Properties

One of the most successful strategies to target Alzheimer’s disease is the development of agents that effectively interact with key enzymes involved in cholinergic dysfunction, especially acetylcholinesterase (AChE). This enzyme terminates the action of acetylcholine neurotransmitters and reduces the information transfer across the synapse [79]. Inhibitory potential of LN against AChE extracted from *B. davidii* was evaluated. Bioautographic assessment on LN and related flavonoids showed that the 4′-OMe group as well as the 7-substituted on the B-ring increased the inhibitory potency [54].

Feng et al. (2017) evaluated the AChE inhibitory potential of LN both in vitro and in vivo. In vitro assays using Ellman’s colorimetric method exhibited an IC_50_ of 3.801 ± 1.149 μM [9]. A molecular docking study showed that the 4′-methoxyl group and the 7-O-sugar moiety of LN might be essential for AChE inhibition. Furthermore, ex-vivo study on mice showed that intraperitoneal administration of LN at doses of 35, 70, and 140 mg/kg decreased the AChE activity on the cortex and hippocampus of mice, where the inhibition effects of LN at the high dose were similar to huperzine A as the positive control (0.5 mg/kg) [80].

**Figure 3:**
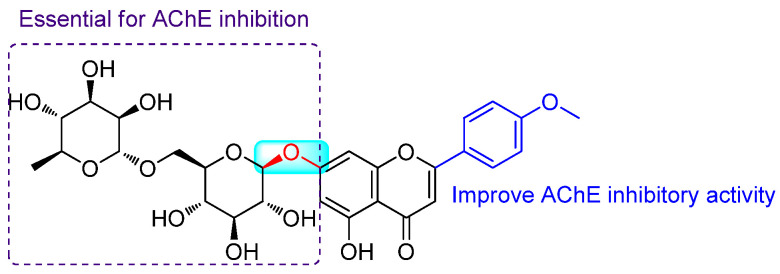
Structure–activity relationship of LN against AChE.

Pan et al. (2019) reported that 16.7 μg/mL and 50 μg/mL of LN (92% pure) had prominent AChE inhibition in zebrafish [81]. In addition, this compound could significantly improve the recovery of dyskinesia in Alzheimer’s disease (animal model). The hydroxyl groups of LN showed strong hydrogen bond interactions with residues Tyr130, Asn85, Trp84, and Asp72 at the anionic subsite of AChE; however, the methoxy flavone segment of LN exhibited π–π interactions with residues Phe331, Trp279, and Phe290 of the peripheral anionic site [81]. The summary of the structure–activity relationship (SAR) of LN against AChE is presented in Figure 3.

### 3.2. Antioxidant Properties

It is well-documented that oxidative stress and neurodegeneration are destructive in central nervous system (CNS) disorders such as Parkinson’s disease and Alzheimer’s disease, and protection of cells from oxidative stress toxicity might be beneficial in the abovementioned diseases. In this regard, Santos et al. exhibited the neuroprotective action of the *V. officinalis* extract in neuroblastoma SH-SY5Y of Parkinson’s disease. To determine the mechanism of action, in silico molecular docking and molecular dynamics evaluations on apigenin, LN, hesperidin, and valerenic acid as the main compounds of *Valeriana* against hub gene transcripts were performed. Specifically, LN fitted strongly to sulfonylurea receptor-1 (SUR1). The ligand mainly interacted with SER 857, accepting one hydrogen bond and donating two. Most likely, LN can relieve the effects of oxidative stress during ATP depletion due to its ability to binding to SUR1 [82].

The high-performance liquid chromatography-electrospray ionization–mass spectrometry (HPLC–ESI–MS) analysis of *C. japonicum* exhibited chlorogenic acid, LN, and pectolinarin as the main compounds. Furthermore, the protective effect of *C. japonicum* on adrenal pheochromocytoma (PC12) cells in vitro and *Caenorhabditis elegans* (in vivo) were also assessed. The cell viability showed a steady increase until 50 μg/mL and then decreased. Pre-treatment of extracts in PC12 cells significantly prevented intracellular ROS accumulation in comparison to the H_2_O_2_ treated control (*p* < 0.05). Under normal growth conditions, treatment with 50 and 100 μg/mL *C. japonicum* extract for 96 h greatly reduced intracellular ROS levels by 37% and 39%, respectively, compared to the control [83].

In the other study, the neuroprotective effect of LN against H_2_O_2_-induced oxidative stress in rat hippocampal neurons was assessed. The results showed that H_2_O_2_ at 400 μM markedly increased the number of apoptotic neurons, while treatment of the neurons with LN significantly reduced the cell death induced by H_2_O_2_ [84].

### 3.3. Sleep Enhancing and Sedative Effect

A set of flavonoid glycosides was evaluated for the sedative, sleeping, and locomotor activity. The following potencies were consequently reported 2S-hesperidin > LN > rutin > diosmin\cong 2S-neohesperidin > gossypin ~ 2S-naringin. The SAR proposed the important role of the 1→6 bond between rhamnose and glucose while changing the bond to 1→2, a remarkable decrease in the activity [85].

Nugroho et al. (2013) reported that LN isolated from the *C. boreale* methanolic extract possessed sedative and sleep-enhancing properties [86]. In detail, 10 and 20 mg/kg LN reduced the latency time for the loss of righting reflex caused by pentobarbital injection and delayed the total duration of sleeping time to around 100 min in mice [86].

### 3.4. Anti-Osteoporosis Activity

The potential application of LN (isolated from *B. officinalis*) in the response against oxidative stress on osteoblastic MC3T3-E1 cells exposed to H_2_O_2_ was evaluated. LN (0.2 µg/mL) significantly increased cell survival, alkaline phosphatase (ALP) activity, collagen content, calcium deposition, and osteocalcin secretion, whereas it decreased the production of the receptor activator of nuclear factor-kB ligand (RANKL), protein carbonyl (PCO), and malondialdehyde (MDA) of osteoblastic MC3T3-E1 cells in the presence of hydrogen peroxide. It was shown that LN exerts antiresorptive actions through the reduction of RANKL and oxidative damage [58]. With more focus toward the antioxidant potential of LN, in another study, the antiosteoporosis activity of *Flos Chrysanthemi indici* on bone loss in ovariectomized mice was evaluated. All isolated compounds including acacetin, apigenin, luteolin, and LN enhanced the differentiation and proliferation of osteoblasts in MC3T3-E1 cells. They also improved the mRNA levels of runt-related transcription factor 2 (RUNX2), osteocalcin (OCN), osteopontin (OPN), and type I collagen. The AKT signaling pathway was also activated in MC3T3-E1 cells by the four compounds [39].

Li et al. (2016) comprehensively evaluated the molecular mechanism pathway of LN on osteoblast differentiation. First, extracted LN from *Flos Chrysanthemi indici* was assessed on MC3T3-E1 cells (a mouse osteoblastic cell line), and next, the osteoprotective effect of LN in mice was evaluated. LN upregulated osteogenesis-related gene expression including that of ALP, OCN, RUNX2, bone sialoprotein (BSP), and type I collagen. Additionally, it was shown that LN enhanced osteoblast proliferation and differentiation in MC3T3-E1 cells dose-dependently through enhanced ALP activity and mineralization of the extracellular matrix by activating the BMP-2/RUNX2 pathway through protein kinase A signaling in vitro, promoting osteoid gene expression and protecting against OVX-induced bone loss in vivo [87].

In addition, a reducing impact of LN on the RANKL-induced macrophage differentiation into multinucleated osteoclasts and osteoclastic bone resorption through reducing lacunar acidification and bone matrix degradation has been demonstrated. Moreover, LN reduced the transmigration and focal contact of osteoclasts to bone matrix-mimicking RGD peptide, which was accomplished by inhibiting the induction of integrins, integrin-associated proteins of paxillin, and gelsolin, cdc42, and CD44 involved in the formation of actin rings [88].

### 3.5. Osteoarthritis Treatment

Osteoarthritis is an age-related joint disease characterized by the degeneration of articular cartilage and chronic pain. Recent studies have confirmed the potential role of anti-inflammatory agents to target osteoarthritis. The LN treatment suppressed lipopolysaccharide (LPS), causing the overproduction of nitric oxide (NO), prostaglandin E2 (PGE2), IL-6, and TNF-α in chondrocyte. In addition, the LPS-stimulated expression of cyclooxygenase-2 (COX-2) and inducible nitric oxide nitrate (iNOS) was decreased by LN pre-treatment. The mechanism of action showed the suppression of Toll-like receptor 4 (TLR4)/myeloid differentiation protein-2 (MD-2) dipolymer complex formation and subsequently intervened in nuclear factor kappa-B (NF-κB) activation [89].

The osteoarthritis mechanism of action of *C. zawadskii* var. *latilobum* extract revealed that the matrix metalloproteinases-1 (MMP-1), MMP-3, MMP-9 and MMP-13 expressions were inhibited by the dose-dependent extract, while expressions of the ECM synthetic genes, COL2A1 and ACAN, and the transcription factor SOX9 were increased to normal condition by the extract treatment dose-dependently. It would be interesting to note that SOX9 is a repressor of ECM-degrading aggrecanases, disintegrin, and metalloproteinase with thrombospondin motifs-4 (ADAMTS-4) and ADAMTS-5, and this extract considerably reduced the levels of these enzymes; it is worth mentioning that these potencies can remarkably be correlated to the LN content of the extract possessing 22.8 mg/g [75].

### 3.6. Ischemia Protection

In the other study, the effect of LN to inhibit ischemia-reperfusion injury was also evaluated. The primary study confirmed the low toxicity of LN (≤30 µM) against normal H9C2 cells. Further assessments showed that LN could protect myocardial tissue from the injury of ischemia-reperfusion related to activation of the Nrf-2 and PI3 K/Akt signaling pathway. Meanwhile, the antioxidative enzymes, regulated by Nrf-2, were enhanced against the oxidative stress caused by hypoxia-reoxygenation. Importantly, with the inhibition of oxidative stress, some proliferation and apoptosis-related proteins such as NF-κB and cytochrome C were adjusted to support the viability of cells [90].

Furthermore, the anti-inflammatory effect of LN during ischemia-reperfusion-acute kidney injuries was assessed. LN inhibited the acute kidney injury in an in vivo ischemia-reperfusion injury model and decreased the expression of interleukin-12 (IL-12) p40 in in vivo and in vitro models. Evaluation on the mechanism of action of LN identified E26 oncogene homolog 2 (ETS2) protein transcription factor for its regulatory action on IL-12 p40 according to microarray analysis and protein–protein interaction. In addition, in silico study showed that the contact area ETS2 is highly conserved and located on a PPI domain of ETS2, which designates that LN may alter the interaction with synergistic proteins in the regulation of IL-12 p40 expression [91].

### 3.7. Anti-Inflammation Activity

Anti-inflammatory assessment of forty-two identified compounds from *Chrysanthemi indici* showed that LN, 3,5-dicaffeoylquinic acid, and luteolin with good biocompatibility could be considered as the important contributors to the anti-inflammatory effect of this plant, which decreased levels of NO, TNF-α, IL-6, and PGE2 in RAW264.7 macrophage cells treated with LPS [92].

In another study, the pelvic inflammatory disease with dampness-heat stasis syndrome was investigated and showed that LN at 8–32 µM can significantly inhibit the NO release in a concentration-dependent manner. Results also confirmed that the inhibitory effects on NO production were not due to the cytotoxicity but strong inhibition of NO production. However, the rapid response of LN on the release of TNF-α upon LPS stimulation for 2 h was not significant [93].

### 3.8. Photoprotective Properties

Acevedo et al. (2005) studied the photoprotective properties of the methanolic extract of *Buddleja scordioides* as well as verbascoside, LN, and linarin peracetate against UV-B induced cell death using *E. coli* as a cell model. Linarin peracetate (2 mg/mL) protected bacteria efficiently with cell death after 125–250 min, while LN reached cell death until 40–80 min. Interestingly, the sun protection factor (SPF) in guinea pigs was 9 ± 0.3 in the LN (2 mg/cm^2^) receiving group, while linarin acetate showed a SPF of 5 ± 0.2. The methanolic extract had the smallest SPF (3 ± 0.09), probably due to the low concentration of the photoprotective compound [61].

Examination of the photoprotective properties of *Buddleja cordata* against UVB-induced skin damage in SKH-1 hairless mice showed that 200 μL of 2 mg/mL extract successfully reduced the redness of UVB irradiation to around 120 within 24 h of UV exposure compared to the untreated group with a redness of 300 [94].

### 3.9. Radioprotection

In another study, LN isolated from *Chrysanthemum morifolium* flowers significantly decreased the IR-induced cell migration and invasion at a concentration of 5 μM in A549 (human lung cancer cells). LN affected cell viability with an IC_50_ value of 282 μM. The mechanism was confirmed via inhibiting NF-κB and IκB-α phosphorylation as well as MMP-9 downregulation [95].

### 3.10. Anti-Apoptosis Potential

The liver injury and hepatic fibrosis caused by the co-treatment with D-galactosamine (GalN)/lipopolysaccharide (LPS) have been extensively approved. Apoptosis is an important cellular pathological process in GalN/LPS-induced liver injury.

In a study conducted by JooKim et al., the cytoprotective mechanisms of LN against GalN/LPS-induced hepatic failure in mice were evaluated. After 6 h of GalN/LPS injection, the serum levels of alanine aminotransferase, aspartate aminotransferase, TNF-α, IL-6, and interferon-γ as well as TLR4 and interleukin-1 receptor-associated kinase (IRAK) expression were significantly elevated.

LN (50 mg/kg) treatment reversed the lethality induced by GalN/LPS via decreasing the levels of TLR4, IRAK, and suppressing the serum release and hepatic mRNA expression of TNF-α, IL-6, and IFN-γ. In the TUNEL assay, in which the apoptotic cells were monitored, LN also suppressed the increase in the number of apoptotic cells and reduced the cytosolic release of cytochrome c and caspase-3 cleavage.

LN administration increased the level of anti-apoptotic Bcl-xL and ratio of p-STAT3/STAT3 protein. Furthermore, LN attenuated the expression of FAS-associated death domain and caspase-8, and reduced the pro-apoptotic Bim phosphorylation induced by GalN/LPS.

These results confirmed the potential properties of LN to suppress TNF-α-mediated apoptotic pathways and pro-apoptotic Bim phosphorylation as well as enhance STAT3 activity and increase anti-apoptotic Bcl-xL levels [33].

### 3.11. Hepatoprotective Function

HPLC-MS analysis of the *Coptis chinensis* inflorescence extract detected 18 flavonoids and alkaloids derivatives including magnoflorine, thebaine, anonarine 5-OH berberine, jateorhizine, columbamine, coptisine, epiberberine, palmatine, berberine, worenine, and LN. Cell viability assessment of *Coptis chinensis* inflorescence extract and LN in HepG2 cells exhibited IC_50_ values of 291.15 and 83.88 μg/mL, respectively. Next, the hepatoprotective function of *C. chinensis* and LN showed the reduction in reactive oxygen species (ROS) generation induced by CCl_4_ in HepG2 cells. LN could also phosphorylate mitogen-activated protein kinases (MAPKs) and upregulate Kelth-like ECH-associated protein (Keap1). The pathways of MAPKs and Keap1 lead to the separation of Keap1 and nuclear factor (erythroid-derived 2)-like 2 (Nrf2). Note that the free Nrf2 transferred to the nucleus and enhanced the expression of phase II detoxification enzymes [71].

### 3.12. Non-Alcoholic Steatohepatitis Effect

Nonalcoholic steatohepatitis (NASH), known as liver inflammation and damage caused by a buildup of fat in the liver, is recognized as a common cause of elevated liver enzymes [96]. Investigations of high-fat high-cholesterol diet in rats showed that LN could suppress the expression of mRNA levels of hepatic inflammation cytokines including monocyte chemotactic protein and TNF-α as well as chemokine ligand 1 (CXCL1). A high dose of LN-extract (60 mg/kg) significantly lowered the serum alanine aminotransferase (ALT) and aspartate aminotransferase (AST) and inhibited the activation of the c-Jun N-terminal kinase (JNK) induced by a high-fat high-cholesterol diet [97].

### 3.13. Anti-Diabetic Effects

The anti-diabetic effects of the *Chrysanthemum zawadskii* extract at different doses (125, 250, and 500 mg/kg body weight) were investigated every day for five or six weeks. The extraction was standardized and showed 1.32 ± 0.22 mg LN/g extract. Subsequently, the extract significantly decreased fasting blood glucose levels in streptozotocin and streptozotocin and high fat diet-induced diabetic models, even at low doses. In addition, glucose tolerance and insulin tolerance were improved by increasing insulin levels and decreasing hemoglobin A1c (HbA1c) levels in serum [98].

Yang-Ji et al. (2016) also demonstrated that the *Chrysanthemum zawadskii* extract could effectively inhibit the lipase and α-glucosidase enzymes to target the diabetic. This potency might well be correlated with the LN content [99].

Similarly, molecular docking, molecular dynamic, conceptual DFT, and pharmacophore mapping studies against α-amylase and α-glucosidase illustrated that LN could be a beneficial preventative and possibly therapeutic agent against diabetes [100].

### 3.14. Analgesic and Anti-Pyretic Properties

MartInez-Vázquez et al. (1996) evaluated the potential analgesic and antipyretic activities of aqueous extract of leaves of *Buddleia cordata* as well as its main compound LN in animal models [101]. The oral administration of an aqueous extract of *B. cordata* and LN showed a dose-dependent antipyretic activity. Aqueous extract and LN (100 mg/kg) remarkably increased the reaction time of mice by 70% and 55% on heat-induced pain, respectively. Similarly, the antipyretic effect of LN was better than that of the aqueous extract in the yeast-induced hyperthermia test. Three hours after the treatment, LN displayed maximal inhibitory effect with the average temperature being reduced by 1.8 °C (50 mg/kg) and 2.0 °C (100 mg/kg), whilst the extract reduced hyperthermia by 1.4 and 1.9 °C at 100 and 200 mg/kg, respectively [101].

### 3.15. Spasmolytic Properties

So far, many studies have approved the remarkable antispasmodic effects of the flavonoids presented in diverse plant species [102,103,104]. LN also showed an acceptable effect investigated by one study. Phytochemical investigation of the hydro-ethanolic extract of *L. japonicus* resulted in the extraction of three flavonoid glycosides named spinosin, LN, and apigenin-7-O-β-D-glucopyranoside as well as four cyclopeptides and nine alkaloids. These compounds were used in the uterine contraction assay. The findings demonstrated that the flavonoid glycosides (spinosin, LN, and apigenin-7-O-β-D-glucopyranoside) at 50 µM inhibited the contraction of the uterine smooth muscle strips significantly; viscerally, cyclopeptides and alkaloids increased contraction of uterine smooth muscle [50].

### 3.16. Treatment of Chronic Venous Hypertension

In a previous experiment, 100 mg/kg/day MPFF (diosmetin, hesperidin, LN, and isorhoifolin) in a chronic venous hypertension animal model showed significant prevention of capillary rarefaction and inflammatory cascade by decreasing the number of sticking leukocytes. MPFF reduced the enlargement of venular diameter as well as maintained venous tone [105].

### 3.17. Anti-Bacterial Activity

Corn mint (*Mentha arvensis*) provides a good source of LN and rosmarinic acid. The methanolic extract inhibited the growth of *Chlamydia pneumoniae* CWL-029 in vitro in a dose-dependent manner. The antichlamydial effect of LN showed complete growth inhibition of strain bacterium *Chlamydia pneumoniae*, and inhibited the growth of strain K7 by >60% at 100 μM. Administration of *M. arvensis* extract (20 mg/kg, 3 days) was able to significantly diminish the inflammatory parameters related to *C. pneumoniae* infection in mice (*p* = 0.019) [47].

### 3.18. Anti-Viral Activity

Virus is a threat to public health due to its high mutation rate and resistance to existing drugs. Recently, the antiviral activity of LN was investigated to develop new antiviral agents. Evaluation of the flavonoid prescription drug baicalin-linarin-icariinnotoginsenoside R1 was assessed on duck virus hepatitis (DVH) caused by duck hepatitis A virus type 1 (DHAV-1). The mentioned drug showed an anti-DHAV-1 ability with T and B lymphocyte-promoting effects. It also inhibited DHAV-1 reproduction by suppressing its adsorption and release. The mechanism of this antiviral effect showed that the drug at 5 µg/mL increased T and B lymphocyte proliferation. Moreover, according to the in vivo study, the drug stimulated total anti-DHAV-1 antibody secretion in ducklings at the dosage of 4 mg per duckling, but had no significant stimulation impact on the IL-2 and IFN-c secretion [106].

In another study, Chen et al. (2017) assessed the baicalin-LN-icariin-notoginsenoside R1 on DHAV-1 as well as its hepatoprotective and antioxidative potencies. Results showed that the DHAV-1 inhibitory rate of this multi-therapy was 69.3% at 20 μg/mL. The survival rate of ducklings treated by 3 mg drug per duckling (once a day for five days) was about 35.5%, which was significantly higher than that of the virus control (0.0%). Additionally, the degree of oxidative stress, the serum MDA, SOD, CAT, and GSH-Px levels at 8 and 54 h were measured and demonstrated a significant reduction compared to the blank and virus groups, which showed the reduction of oxidative stress in the infected duck [107].

**Figure 4:**
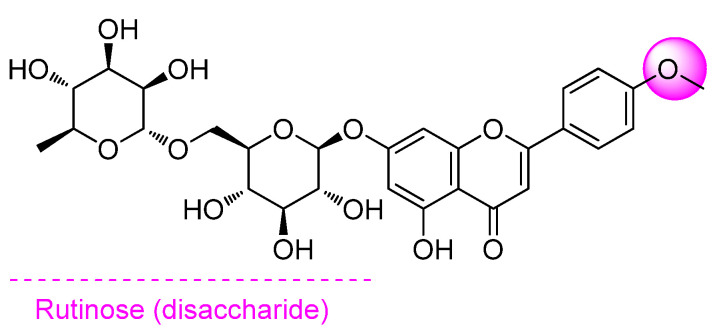
Structure–activity relationship of LN against HIV.

Human immunodeficiency virus (HIV) is an infection that attacks the body’s immune system, specifically the white blood cells called CD4. The development of anti-HIV-1 drugs has gained much attention nowadays [108]. It has been shown that human γδ T cells (lymphocytes) consist of Vδ1-TCR-expressing Vδ1+ T cells and Vδ2-TCR-expressing Vδ2+ T cells, which play pivotal roles in bridging innate and adaptive immunity. It was proposed that stimulation Vδ1+ T cells may constitute a new class of anti-HIV drugs, targeting the mucosal compartment to suppress the R5-type of HIV-1. Yonekawa et al. (2019) reported that LN at 100 μg/mL and some flavonoid glycosides, which have both rutinose at the A ring and methoxy substitution at the B ring, can activate host Vδ1+ T cells in HIV patients and can contribute to limiting the R5-type of HIV-1 replication. LN stimulated PBMC-derived Vδ1+ T cells to secrete chemokines MIP-1α, MIP-1β, and RANTES and cytokines such as IL-5 and IL-13, which may improve the immune system [109]. Figure 4 exhibits the structure–activity relationship of LN against HIV.

In another study, virtual screening on Chinese medicinal compounds was applied to discover novel natural drugs against the influenza A virus using Naïve Bayesian classifiers, and mt-QSAR models. In the selected set, LN exhibited a significant reduction in TNF-α expression to around 40 pg/mL compared to the control group with ~80 pg/mL, whereas it may regulate the expression of cytokines and chemokines, which represent direct and indirect suppression of influenza A [110].

### 3.19. Anti-Cancer and Anti-Proliferative Activity

Cancer is one of the major causes of death worldwide, affecting more than 14.1 million people worldwide [111]. Over the past few years, attention has been paid to find potent natural products as anticancer therapeutic agents [79,112,113].

Flavonoids are known to be one of the most popular groups of bioactive phytochemicals with anticancer activity; however, limited study has been conducted to evaluate the activity of LN as anticancer agents [79].

The methanolic extract of *Chrysanthemum indicum* and purified LN exerted anti-proliferative activity against human non-small cell lung cancer cells via suppression of Akt activation and induction of cyclin-dependent kinase inhibitor p27Kip1, as evidenced by cell cycle analysis and treatment with LY294002. These findings may indicate the anticancer potential of LN as the core functional constituent of *C. indicum* [114].

Glioma is the most common form of malignant brain cancer with a high mortality rate in humans. NF-κB activity is a common phenomenon in various cancers, resulting in abnormal cell proliferation, malignant transformation, or resistance to cell death. Previously, the anti-cancer role of LN in glioma was tested in vitro and in vivo. LN suppressed glioma cell proliferation and migration by inducing apoptosis, which was through reducing the cell cycle-related signals including survivin, p-Rb, and cyclin D1, while promoting p21, Bax, caspase-3, and poly (ADP-ribose) polymerase (PARP) activation. LN also showed an increase in P53 as an essential tumor suppressor. Moreover, it reduced cellular proliferation of glioma through p53 upregulation and NF-κB/p65-downregulation, thereby inhibiting glioma cell growth [115].

The cytotoxicity of *Jatropha pelargoniifolia* loaded chitosan nanoparticles against A549 human lung adenocarcinoma cells (IC_50_ = 13.17 µM) was higher than that of the free extract (IC_50_ = 25.16 µM) and comparable to that of methotrexate (IC_50_ = 11.84 µM) as an anticancer drug [116].

Oral squamous cell carcinoma is characterized by overexpression of Akt1 (RAC-alpha serine/threonine-protein kinase) and Akt2 (RAC-beta serine/threonine-protein kinase). It was reported that Akt1 and Akt2 inhibitors can lead to oral squamous cell carcinoma treatment with no affinity toward monoamine oxidase B (MAOB). In silico studies introduced LN as inhibitors of Akt1 and Akt2 with strong binding affinities of 11.5 kcal/mol and 11.1 kcal/mol, respectively, with no affinity toward MAOB, which can be an ideal candidate for oral squamous cell carcinoma treatment [117].

### 3.20. Negative Biological Results of LN

#### 3.20.1. Estrogenic Activity

The estrogenic activity of six chemical constituents (apigenin, hispidulin, cirsimaritin, cirsimarin, pectolinarin, and LN) isolated from *Cirsium japonicum* on MCF-7 cells was assessed. Among them, hispidulin and cirsimaritin showed strong estrogen receptor transactivation, while the rest of the compounds had weaker or relatively no effects. The SAR confirmed that estrogen receptor transactivation increases as the number of –OH groups in the flavonoid structure increased [19].

#### 3.20.2. Anti-Fungal Effect

Combined chromatographic techniques were implemented in the phytochemical analysis of *Lippia rubella*, leading to the isolation of several compounds such as lippiarubelloside A and lippiarubelloside B, verbascoside as well as LN. Inhibitory evaluation of LN against some fungal strains such as *Candida albicans* (ATCC 10231) and *Candida parasilopsis* (ATCC 22019) asserted no significant activity (MIC >125 μg/mL), and moderate effects against *Cryptococcus neoformans* and *Cryptococcus neoformans* (MIC: 125 μg/mL) [73].

#### 3.20.3. Anti-Depressant Properties

Depression is a mental health disorder characterized by loss of interest, pleasure, with feelings of sadness, low self-worth, and tiredness, which disturbed sleep or appetite, leading to suicide in severe cases. The exact mechanism of depression is still unknown, and most of the antidepressants act as inhibitors of intracellular monoamine (exp, norepinephrine) reuptake. Additionally, it has been shown the gamma-aminobutyric acid (GABA) levels as well as cortical GABAA receptors decreased in patients with depression.

In this regard, the norepinephrine reuptake of *Cirsium japonicum* and its major constituents (linarin, pectolinarin, chlorogenic acid, luteolin) were evaluated. *Cirsium japonicum* showed an antidepressant effect by significantly reducing the immobile behavior of mice in the forced swimming test, without enhancing locomotor activity in the open-field test. In addition, the *C. japonicum* extract had no effect on monoamine uptake while significantly promoting Cl^–^ ion influx in human neuroblastoma cells and modulating the GABAA receptor. Further evaluation showed that among the major constituents of the *C. japonicum* extract, only luteolin produced antidepressant activity as a positive modulator of the GABA-mediated Cl^−^ ion channel complex and LN was almost inactive [118]. Results showed that the antidepressant effect of *Cirsium japonicum* could be due to the luteolin constituent.

## 4. Perspectives

### Anti-SARS-CoV-2 (COVID-19) Effect

Severe acute respiratory syndrome-coronavirus 2 (SARS-CoV-2) is a RNA airborne virus infection known as the pathogen responsible for coronavirus disease 2019 (COVID-19) [119]. Millions of COVID-19 patients have been reported thus far; however, there is no concrete evidence on the effectiveness and safety of the specific treatment against SARS-CoV-2 [120,121]. One area that has been affected immensely is the investigation of natural remedies as medications and/or supportive therapies to treat patients with COVID-19 infection. Mostly, antiviral drugs directly target the infecting pathogen to halt its development [122]. In the case of SARS-CoV-2, the influence of active substances of medicinal plants were surveyed in inhibiting four important druggable targets including S and N proteins, 3CLpro, and RdRp. RdRp controls the replication of SARS-CoV-2 while 3CLpro is the main protease of the virus. Moreover, N and S proteins are responsible for SARS-CoV-2 assembly and attachment, respectively. Molecular docking outcomes of the study revealed that LN, amentoflavone, (-)-catechin gallate, and hypericin had an affinity for these basic proteins, which possess an effective role in SARS-CoV-2 infection [123].

## 5. Conclusions

Investigation of plant secondary metabolites with valuable impacts on human health is an attractive and broad research area. Flavonoids, a large family of phenolic compounds due to their pivotal therapeutic effects, have been the subject of many studies. Nowadays, their diverse derivatives are widely consumed as dietary supplements. Although the most renowned flavonoids (i.e., apigenin, luteolin, hispidulin, kaempferol, myricetin, quercetin, naringenin, etc.) are aglycosylated [124], the glycosylated forms are also of interest. It is believed that the glycosylation of flavonoids can lead to the development of their biological features by reducing the probable toxicity and increasing their bioavailability [125].

The present context overviewed a very promising but not well-investigated glycosylated flavone named LN. From the phytochemical viewpoint, the plant genus *Cirsium*, *Micromeria*, *Buddleja*, and *Chrysanthemum* are the major natural sources of LN. This compound demonstrated promising bioactivities through the studies carried out in vitro and in vivo. The encouraging properties of LN have been shown through osteoblast proliferation and differentiation with high anti-arthritis and antiosteoporosis potencies; however, its effect on the treatment of CNS disorders have also been pointed out.

Further phytochemical investigations of different natural sources leading to the isolation and identification of LN as well as exploring the optimized extraction methods can support the implementation of its bioactivity assessments. Complementary biological and pharmacological evaluations (particularly toxicity and clinical trials) of LN and its derivatives are proposed in future in order to develop potential natural-based drugs/supplements with the least side effects.

## Data Availability

Not applicable.

## Supplementary Materials

The followings are available online at https://www.mdpi.com/article/10.3390/ph14111104/s1, Table S1: Isolation and identification of linarin from plant species; Table S2: Identification and characterization of linarin from plant species; Table S3: Biological properties of linarin. References [126,127,128,129,130,131,132,133,134,135,136,137,138] are cited in the supplementary materials.
