# Supplementary material for: Linarin, a Glycosylated Flavonoid, with Potential Therapeutic Attributes: A Comprehensive Review
Source: Pharmaceuticals (Basel). 2021 Oct 29;14(11):1104. doi: 10.3390/ph14111104 (PMC8621830; doi:10.3390/ph14111104)
Supplement: Supplementary file 1 [file pharmaceuticals-14-01104-s001.zip › pharmaceuticals-1425982-SI.pdf]

Table S1. Isolation and identification of linarin from plant species.

Family	Plant species	Subjected Plant Part/ Soluble Extract	Method of Isolation/Purification	References
Asteraceae	*Cirsium arvense*	AP/ MeOH	CC [$CHCl_3$–MeOH 1:0, 0:1]	[13]
	Cirsium arvense subsp. vestitum	R/ nd	CC [EtOH-DCM 1:1, 1:3]	[14]
	Cirsium japonicum	AP/ nd	CC, SLH	[16]
	Cirsium japonicum var. *ussuriense (Regel) Kitam. ex Ohwi*	AP/ MeOH	CC [$CHCl_3$–MeOH-H_2O 25:8:5]	[20]
	Cirsium setidens	F/ MeOH	CC [H_2O-MeOH 0:100 to 100:0]	[22]
	Cirsium setosum (Willd.) MB.	nd/ EtOH	CC [EtOH 20%, 40%, 60%, 80%, 100%]	[25]
	Chrysanthemum boreale	F/ MeOH	CC [$CHCl_3$–MeOH-H_2O 70:30:10]	[26]
		F/ MeOH	CC [MeOH-$CHCl_3$ 5-9%]	[27]
	Chrysanthemum morifolium Ramat.	F/ EtOH (95%)	CC [$CHCl_3$- MeOH 10:0 to 1:9]	[28]
	Chrysanthemum Indicum	nd/ EtOH (80 %)	HSCCC [HEX-$CHCl_3$-MeOH-H_2O 0.5:4:3:2 - $CHCl_3$-MeOH-H_2O 4:3:2]	[39]
		F/ MeOH	CC [EtOAc-MeOH-H_2O 100:10:7]	[33]
		AP/ DCM	CC [DCM-MeOH 20:0 to 1:1]	[35]
	Chrysanthemum zawadskii var. *latilobum* Kitamura	H/ MeOH	recrys. (pyridine and MeOH)	[38]
		AP/ EtOAc	CC [EtOAc- MeOH], recrys. (EtOAc-MeOH)	[37]
	Artemisia capillaris	WP/ MeOH	CC [CH_2Cl_2-MeOH 20:1]	[43]
	Picnomon acarna	AP/ PET, Bz, CHCl3, and MeOH	CC [MeOH]	[44]
Campanulaceae	*Lobelia chinensis* Lour.	H/ MeOH	Semi-preparative HPLC [H_2O–MeOH 5–100%]	[66]
Gentianaceae	*Exacum macranthum*	WP/ PET, CHCl3 and MeOH	recryst., hydrolysis [HCl-MeOH (6 %)]	[65]
Lamiaceae	*Mentha arvensis*	F/ MeOH (80 %)	CC [$CHCl_3$-MeOH-H_2O (2.5% AA) 65:35:5]	[46]

	Mentha spicata	AP/ EtOAc	CC [HEX, HEX-EtOAc, EtOAc, EtOAc-MeOH, MeOH]	[48]
	Mentha piperita	AP/ EtOAc	CC [HEX, HEX-EtOAc, EtOAc, EtOAc-MeOH, MeOH]	[48]
	Mentha villosonervata	AP/ EtOAc	CC [HEX, HEX-EtOAc, EtOAc, EtOAc-MeOH, MeOH]	[48]
	Dracocephalum peregrinum	AP/ *n*-BuOH	polyamide [EtOH:H_2O 25:75-50:50-100:0]; SLH [MeOH:H_2O (80:20)]	[49]
	Leonurus japonicas	AP/ EtOH (95 %)	CC [DCM-MeOH 100:1 – 0:100]	[50]
	Calamintha glandulosa	L/ MeOH (80%)	Semi-preparative HPLC [H_2O-ACN 50 to 0%]	[52]
	Ziziphora clinopodioides	H/ MeOH	nd	[53]
Malvaceae	*Bombax malabaricum DC.*	F/ HEX and MeOH	polyamide [H_2O–MeOH]; PC [*n*-BuOH, H_2O (15% AA) 4:1:5]	[68]
Poaceae	*Avena sativa*	nd/ EtOH (95%)	CC [EtOH-H_2O 0:100, 30:70, 60:40, 100:0]	[69]
Ranunculaceae	*Thalictrum aquilegifolium*	WP/ MeOH	CC [$CHCl_3$-MeOH 20:1]; recrys. (pyridine-H_2O)	[70]
Rutaceae	*Zanthoxylum affine*	AP/ HEX	CC [HEX-acetone 9:1, 85:15]	[72]
Scrophulariaceae	*Buddleja davidii*	L/ MeOH	CPC [$CHCl_3$-MeOH-H_2O 45:33:22]	[126]
	Buddleja asiatica Lour.	WP/ EtOH (95%)	CC [EtOAc-MeOH-H_2O 9:1:0.1, 8:2:0.2,7:3:0.3]	[55]
	Buddleja cordata Kunth	WP/ MeOH	CC [$CHCl_3$-MeOH]	[57]
	Buddleja officinalis	F/nd	nd	[58]
		F/ MeOH	CC [$CHCl_3$-MeOH-H_2O 5:1:0.1]	[60]
	Buddleja scordioides	AP/ HEX, MeOH, EtOAc	CC [$CHCl_3$-MeOH 19:1, 9:1, 8:2]	[61]
	Linaria japonica	WP/ MeOH	CC [$CHCl_3$-MeOH 1:0 to 3:1], recrys. (MeOH), DCCC [$CHCl_3$-MeOH-H_2O-*n*-PrOH 45:60:40:10]	[62]

	Linaria kurdica subsp. eriocalyx	WP/ *n*-BuOH	CC [CH_2Cl_2-MeOH 9:1, 8:2, 7:3], SLH [MeOH]	[64]
Valerianaceae	*Valeriana officinalis* L.	R/ EtOH (70 %)	CC [MeOH $CHCl_3$ 20:80]	[126]
	Valeriana wallzchzz DC.	nd	PTLC [EtOAc-MeOH-H_2O 200:33:27]	[127]
Verbenaceae	*Lippia rubella*	AP/ EtOH (95 %)	CCC [HEX-EtOAc-MeOH 4:2:05]	[73]

AcOH: AP: aerial part, B: bud, Bz: benzene, CC: column chromatography on silica gel, CCC: counter-current chromatography, $CHCl_3$: chloroform, CPC: centrifugal partition chromatography, DCCC: droplet countercurrent chromatograph, DCM: dichloromethane, ETH: ether, EtOAc: ethyl acetate, EtOH: ethanol, F: flower, FA: formic acid, GAA: glacial acetic acid, H: herb, H_2O: water, HCl: hydrochloric acid; HCO_2H: formic acid, HSCCC: high-speed counter-current chromatography, ACN: acetonitrile, MeOH: methanol, *n*-BuOH: *n*-butanol, *n*-PrOH: *n*- propanol, HEX: *n*-hexane, nd: not determined, PC: paper chromatography, PET: petroleum ether, PTLC: preparative thin layer chromatography, R: root, recrys.: recrystallization, SL: solid-liquid extraction, SLH: Sephadex® LH-20, WP: whole part.

Table S2. Identification and characterization of linarin from plant species.

Family	Plant species	Subjected Plant Part/ Soluble Extract	Analytical Instrument	Eluent System for Chromatography	Quantity	References
Asteraceae	*Cirsium arvense*	AP/ MeOH	HPLC-UV	nd	nd	[13]
	Cirsium arvense subsp. vestitum	R/ nd	HPLC-MicroTOF-Q	nd	15 mg	[14]
	Cirsium canum (L.)	F/<br>MeOH (50%)<br>MeOH (80%)<br>MeOH (100%)<br>DCM<br>Acetone<br>EtOAc	HPLC-DAD	H_2O (1% AA)-MeOH [2 to 75%]	121.75 µg/g<br>116.12 µg/g<br>44.13 µg/g<br>1.94 µg/g<br>10.51 µg/g<br>8.94 µg/g	[15]
	Cirsium japonicum	AP/ MeOH	HPLC-MS	H_2O-ACN (0.025% TFA) [80:20 to 5:95]	0.26–1.15 mg/100 g	[16]
	Cirsium japonicum var. maackii	nd/ EtOH	HPLC	ACN-H_2O (0.5% AA)	nd	[19]
	Cirsium japonicum var.ussuriense (Regel) Kitam. ex Ohwi	AP/ MeOH	nd	nd	nd	[20]
	Cirsium rivulare	L/ MeOH	HPLC	ACN-H_2O (1% AA) [10:90, 30:70, 10:90]	170 mg/g	[21]
		F/ MeOH	HPLC	ACN-H_2O (1% AA) [10:90, 30:70, 10:90]	20 mg/g	
	Cirsium setidens	F/ MeOH	HPLC	H_2O (0.1% FA)-ACN	120.3 mg/g	[22]
	Cirsium setosum (Willd.) MB.	AP/ nd	HPLC	H_2O-ACN (0.025% TFA) [80:20 to 5:95]	0.3-2 mg/100 g	[16]
			HPLC-MS	H_2O-ACN (0.1% TFA) [80:20 to 5:95]	nd	

	AP/ nd	LC	H_2O (0.5% AA)-ACN [87:13, 75:25, 60:40, 90:10, 80:20]	1.920-4.315 mg/g	[23]
		LC-MS	H_2O (0.5% AA)-ACN [35 to 50%]		
	nd/ EtOH (70 %)	LC	MeOH-H_2O (0.1% AA) [35:65, 60:40, 70:30, 86:14, 95:5]	1.524 - 21.213 mg/ g	[24]
		HPLC	MeOH-H_2O [50:50]		
	nd/ EtOH	UPLC–MS	H_2O (0.1% FA)-ACN (0.1% FA) [90:10, 70:30, 70:30, 45:55, 5:95]	nd	[25]
Hemistepta lyrata	F/ MeOH	HPLC-UV	H_2O (TFA 0.05%)-MeOH+ACN (60:40) (+TFA 0.05%) [85:15, 35:65, 0:100, 85:15]	0.06-4.26 mg/g	[26]
Chrysanthemum morifolium Ramat.	F/ EtOH (95%)	HPLC-DAD-ESI/MS	H_2O (0.1% FA)-ACN (0.1% FA) [85:15, 65:35, 40:60, 5:95, 85:15]	0.117-0.582 mg/g	[28]
Chrysanthemum Indicum	F/ MeOH	HPLC/DAD	H_2O (0.05% H_3PO_4)-ACN [88 to 74%]	nd	[31]
	F/ EtOH (80 %)	HPLC-UV	H_2O-ACN (0.1% H_3PO_4) [15-80%]	32.8 mg/g	[39]
	AP (L,S,F)/ DCM<br>L<br>S<br>F	UPLC	ACN-H_2O (0.1% FA) [5:95, 15:85, 25:75, 40:60, 55:45, 100:0, 5:95]	 1.47 g/100g<br>0.65 g/100 g<br>0.64 g/100g	[35]
	WP (R,S,L,F,FB)/ MeOH	HPLC-MS	H_2O (0.1% FA)-ACN (0.1% FA) [72:28 to 28:72]	R: 0.364 µg/mg FW<br>S: 0.133 µg/mg FW<br>L: 0.070 µg/mg FW<br>F: 0.052 µg/mg FW<br>FB: 0.064 µg/mg FW	[34]
	nd/ EtOH (75 %)	HPLC-UV	MeOH-H_2O (+AA) [50:49.95:0.05]	55.68-2.08 %	[36]
		HPLC-DAD	MeOH-H_2O (0.2% AA) [40:60, 50:50, 100:0]		
	F/ MeOH	HPLC-UV	H_2O (0.4% FA)-ACN (0.4% FA) [100:0, 90:10, 80:20]	nd	[32]
	F and B/ EtOH (95 %)	HPLC-DAD	ACN-H_2O (0.1% FA) [5:95, 15:85, 30:70, 50:50]	48.3 mg/g	[30]
	F/ EtOH	HPLC-UV	H_2O (0.1% AA)-MeOH [52:48]	28.85-88.11%	[40]

		nd/ MeOH (60%)	HPLC-DAD-MS	ACN (0.1% FA)-H_2O (0.1% FA) [10:90, 26:74, 65:35, 100:0]	nd	[41]
		nd/ MeOH	HPLC-DAD	ACN-H_2O (1% AA) [10:90, 30:70, 10:90]	14.6-15.3 μg/g	[42]
Campanulaceae	*Lobelia chinensis* Lour.	H/ MeOH	LC-MS	MeOH-H_2O [5:95 to 100:0]	nd	[66]
			HPLC-DDA-MS	ACN-H_2O [5:95, 30:70, 70:30]	nd	
Lamiaceae	*Mentha arvensis*	F/ MeOH (80 %)	HPLC-UV	ACN-H_2O [5:95 to 95:5]	6 %	[46]
		AP/ MeOH (80 %)	HPLC-DAD<br>UPLC-ESI/Q-TOF/MS	MeOH-H_2O (+AA) (10:88:2); MeOH-H_2O (+AA) (90:8:2) [95:5, 90:10, 75:25, 65:35, 0:100]<br>ACN-H_2O (0.1% FA) [10 to 95% ACN]	nd	[47]
	Mentha haplocalyx	nd	HPLC-MS/MS	ACN-H_2O (0.3% FA) [5:95, 15:85, 19.5:80.5, 22:78, 30:70]	nd	[76]
	Mentha spicata	AP/ EtOAc	HPLC-UV	ACN-H_2O (2.5% FA) [15:85, 25:75, 100:0]	2.79-42.21 mg/g	[48]
	Mentha piperita	AP/ EtOAc	HPLC-UV	ACN-H_2O (2.5% FA) [15:85, 25:75, 100:0]	0.04-0.27 mg/g	[48]
	Mentha villosonervata	AP/ EtOAc	HPLC-UV	ACN-H_2O (2.5% FA) [15:85, 25:75, 100:0]	0.26-17.93 mg/g	[48]
	Acinos arvensis ssp. Villosus	L/ MeOH (80%)	HPLC-UV	H_2O-MeOH [50:50, 40:60, 0:100]	nd	[52]
	Acinos hungaricus	L/ MeOH (80%)	HPLC-UV	H_2O-MeOH [50:50, 40:60, 0:100)	nd	[52]
	Calamintha officinalis	L/ MeOH (80%)	HPLC-UV	H_2O (0.85% H_3PO_4)-ACN-MeOH [100:0:0, 87:13:0, 72:19:9, 12:70:18, 5:80:15, 100:0:0]	0.27 mg/g	[51]
	Calamintha glandulosa	L/ MeOH (80%)	HPLC-UV	H_2O-MeOH [50:50, 40:60, 0:100]	nd	[52]
	Micromeria albanica	L/ MeOH (80%)	HPLC-UV	H_2O-MeOH [50:50, 40:60, 0:100]	nd	[52]
	Micromeria cristata	L/ MeOH (80%)	HPLC-UV	H_2O-MeOH [50:50, 40:60, 0:100]	nd	[52]
	Micromeria dalmatica	L/ MeOH (80%)	HPLC-UV	H_2O-MeOH [50:50, 40:60, 0:100]	nd	[52]

	Micromeria Juliana	L/ MeOH (80%)	HPLC-UV	H_2O-MeOH [50:50, 40:60, 0:100]	nd	[52]
	Micromeria thymifolia	L/ MeOH (80%)	HPLC-UV	H_2O-MeOH [50:50, 40:60, 0:100]	nd	[52]
	Satureja cuneifolia	L/ MeOH (80%)	HPLC-UV	H_2O-MeOH [50:50, 40:60, 0:100]	nd	[52]
	Satureja kitaibelii	L/ MeOH (80%)	HPLC-UV	H_2O-MeOH [50:50, 40:60, 0:100]	nd	[52]
	Satureja montana ssp. Montana	L/ MeOH (80%)	HPLC-UV	H_2O-MeOH [50:50, 40:60, 0:100]	nd	[52]
	Ziziphora clinopodioides	nd/ EtOH (70%)	UPLC-Q-TOF-MS	H_2O (0.1% FA)-ACN [5 to 100 ACN]	nd	[77]
		H/ MeOH	RP-RRLC	H_2O (1% AA)-MeOH [85:15, 60:40, 45:55]	3.15-20.55 mg/g	[53]
Ranunculaceae	*Coptis chinensis*	F/ EtOH (70 %)	HPLC-MS	H_2O (0.1% FA) [85:75 to 75:55]	nd	[71]
Rutaceae	*Zanthoxylum affine*	AP/ HEX, Acetone and MeOH	HPLC–Q-TOF-MS	H_2O (0.01% FA)-MeOH [80:20, 50:50, 10:90, 80:20]	nd	[72]
Scrophulariaceae	*Buddleja davidii and B. nitida*	L/ MeOH	LC-MS/MS	H_2O-MeOH [15 to 100% MeOH]	nd	[54]
	Buddleja cordata Kunth	L/ EtOH (70 %)<br>White callus/ EtOH (70 %)<br>Green callus/ EtOH (70 %)<br>R/ EtOH (70 %)	HPLC-DAD	H_2O (2% AA)-ACN [100:0, 75:25, 0:100, 100:0]	41.81 ± 5.21 mg/g<br>2.12 ± 0.21 mg/g<br>2.34 ± 0.13 mg/g<br>3.01 ± 0.5 mg/g	[56]
	Buddleja officinalis	F/ EtOH (70 %)	UHPLC-LTQ-Orbitrap	H_2O (0.1% FA)-ACN [90:10, 60:40, 10:90, 90:10]	nd	[59]
	Linaria vulgaris	nd/infusion	HPLC-UV	H_2O (5% FA)-MeOH [50:50, 40:60]	3840.9 mg/kg	[63]
Valerianaceae	*Valeriana edulis*	nd/ MeOH	HPLC-DAD	H_2O-ACN (50% in MeOH, 1:1) [98:2, 83:17, 50:50, 0:100]	<0.002%	[78]

	Valeriana officinalis L.	nd/ MeOH	HPLC-DAD	H_2O-ACN (50% in MeOH, 1:1) [98:2, 83:17, 50:50, 0:100]	0.002%	[78]
	Valeriana jatamansi	nd/ MeOH	HPLC-DAD	H_2O-ACN (50% in MeOH, 1:1) [98:2, 83:17, 50:50, 0:100]	0.24%	[78]
	Valeriana procera	nd/ MeOH	HPLC-DAD	H_2O-ACN (50% in MeOH, 1:1) [98:2, 83:17, 50:50, 0:100]	0.07%	[78]
	Valeriana sitchensis	nd/ MeOH	HPLC-DAD	H_2O-ACN (50% in MeOH, 1:1) [98:2, 83:17, 50:50, 0:100]	0.03%	[78]

AA: acetic acid, ACN: acetonitrile, AP: aerial part, DCM: dichloromethane, B: bud, DAD: diode array detector, ESI: electrospray ionization, EtOAc: ethyl acetate, EtOH: ethanol, F: flower, FB: flower bud, FW: fresh weight, H: herb, H_3PO_4: phosphoric acid, H_2O: water, FA: formic acid, HPLC: high-performance liquid chromatography, L: leaf, LC: liquid chromatography, MeOH: methanol, MS: mass spectrometry, NHEX: *n*-hexane, nd: not determined, QTOF: quadrupole and a time-of-flight analyzer, R: root, RP: reverse phase, RRLC: rapid-resolution liquid chromatography, S: stem, TFA: trifluoroacetic acid, UPLC: ultra-performance liquid chromatography, UV: ultraviolet-Visible, WP: whole part.

Table S3. Biological properties of linarin.

Activity	Isolated from plant/prepared	Measure of activity	Assay	Experimented media	Positive controls	Activity of controls	Cell lines/Strain/Model	References
**Acetylcholinesterase-Inhibitory**	*Buddleja davidii*<br>MeOH ex. L	D.L.: 10 ng	Bioautographic (TLC)	In vitro	Huperzine A	D.L.: 1 ng	-	[54]
	purchased	IC50: 3.801 ± 1.149 μM (in vitro)<br>I.D.: 140 mg/Kg	Ellman's colorimetric	In vitro<br>Ex vivo	Huperzine A	I.D.: 0.5 mg/kg	Mouse brain (in vitro)<br>cortex and hippocampus of mice (ex vivo)	[80]
	prepared	DRR: 76.8% (at C: 16.7 μg/mL)<br>DRR: 88.0% (at C: 50 μg/mL)	molecular docking simulation	In vivo	Donepezil	DRR: 79.3% (at C: 8 μM)<br>DRR: 22.6% (at C: 5.6 μg/mL)	Zebrafish	[81]
**Analgesic (Antinociceptive)**	*Buddleia cordata*<br>Aqueous ex.	A.W.: 18.6 ± 3.3; P: 38% (at D: 25 mg/kg)<br>A.W.: 16.6 ± 2.3; P: 45% (at D: 50 mg/kg)<br>A.W.: 14.5 ± 1.0; P: 52% (at D: 100 mg/kg)<br>A.W.: 12.8 ± 3.8; P: 57% (at D: 200 mg/kg)	Writhing test	In vivo	Morphine sulphate<br>acetylsalicylic acid	A.W.: 4.7 ± 0.6; P: 90% (D: 1.15 mg/kg)<br>A.W.: 10.6 ± 2.7; P: 65% (D: 200 mg/kg)	mice	[101]
		V: 55% (at D: 100 mg/kg)	Hot-plate test		Morphine sulphate<br>acetylsalicylic acid	V: 68% (D: 3 mg/kg)<br>V: 8% (D: 100 mg/kg)		
	Buddleia cordata<br>Aqueous ex.	ED_{50}: 89.0 mg/kg	Hot-plate test	In vivo	acetylsalicylic acid	ED_{50}: 28.3 mg/kg	mice	[128]

**Anti-cancer**	purchased	I: 3.1-fold for NFATc1 gene (at C: 0 µg/mL)<br>I: 2.2-fold for NFATc1 gene (at C: 0.1 µg/mL)<br>I: 2.2-fold for NFATc1 gene (at C: 1 µg/mL)<br>I: 1.8-fold for NFATc1 gene (at C: 10 µg/mL)<br><br>I: 3.8-fold for TRAP gene (at C: 0 µg/mL)<br>I: 2.6-fold for TRAP gene (at C: 0.1 µg/mL)<br>I: 2.3-fold for TRAP gene (at C: 1 µg/mL)<br>I: 2.1-fold for TRAP gene (at C: 10 µg/mL)	nd	nd	nd	nd	Bone marrow macrophages	[129]
	Buddleja officinalis	Collagen: 85~105%<br>C.V: 45~100%<br>ALP activity: 85~108%<br>M: 80~100%<br>MDA: 100~200%<br>PCO: 100~130%<br>OCN: 55~100%<br>RANKL: 100~250%	MTT	In vivo<br>In vitro	H_2O_2	Collagen :44.79 ± 1.11 lg/10^6<br>ALP: 3.61 ± 0.07 U/10^6<br>M:1.11 ± 0.01<br>POC and MDA:2.78 ± 0.16 and 5.34 ± 0.09 nmol/mg<br>OCN: s 6.84 ± 0.30 ng/10^6<br>RANKL: 14.33 ± 0.67 pg/106	cell	[58]
	Chrysanthemum zawadskii	RHR: (60 % and 40 %)	_	_	SD	_	_	[130]

	hydro-methanolic<br>(80%) ex./AP							
	Dendranthema zawadskii var. *latilobum*<br>EtOAc ex./AP	–	–	–	–	–	–	[37]
	Chrysanthemum indicum	CP: (1~1.5)<br>CV: (80~95)(%)	–	–	FBS	FBS: 10 %	A549 Human lung cancer	[32]
	Chrysanthemum indicum	TNF-α: (200 pg/ml)<br>IL-6: (500 pg/ml)<br>IFN-γ: (90 pg/ml)<br>TNF-α mRNA: (150 pg/ml)<br>IL-6 mRNA: (180 pg/ml)<br>IFN-γ mRNA: (0.3 pg/ml)<br>TLR4: 1.2<br>IRAK: 2<br>Cytosolic: 4<br>FADD protein: 0.8<br>caspase-3: 0.2<br>caspase 8: 0.5<br>P_Bim$_{LE}$: 0.5<br>P_Bim$_{L}$: 1.8<br>Bcl-Xl: 2.8<br>P_ STAT3: 2.1	cycle threshold	–	ALT<br>AST	ALT: 31.37±8.7 U/L<br>AST: 39.27±2.8	Mouse liver	[33]
	Flos Chrysanthemi Indici	ALP activity :(13,15)(OD/mg)<br>COL-I:(5,7)(OD/mg)<br>OCN:(4,6)(OD/mg)<br>OPN: (4,5)(OD/mg)<br>Runx2:(5,6)((OD/mg)	HSCCC	In vitro<br>In vivo		Mineralization: 150%<br>P-AKT/AKT: 8%<br>Runx2: 7 %	mice	[39]

	CCK-A:(1.2,1.5,2)%						
Cirsium arvense subsp. *Vestitum*	–	–	in vitro	BHT, BHA	I C6: (-10~20)%<br>I HeLa: (0~-5)%	mice	[14]
Flos Chrysanthemi Indici	ALP: (1~7 day)(10~20)(OD/mg)<br>M:(110~270)%	–	In vivo<br>In vitro	–	Collagen: 2.8%<br>ALP: 4%<br>OCN:2.7%<br>BSP:3%<br>RUNX2:4.5%<br>BMP-2: (150~280)%<br>SMAD1/5: (210~400)%	mice	[87]
Flos *Chrysanthemi Indici*	DEVDase activity: 0.12<br>Colony fotmation:20%<br>Oligo nucleosome:4 unit/flod<br>Apoptotic cell:75%<br>Mitochondria members:40%<br>cytochrome c (Cyto-c): (02.~,5.6) flod of con<br>ROS production:40%<br>JNK activation: (4,4.5)flod of con	MTT	In vivo<br>In vitro	KI-67 and TUNEL	tumor weight: 1 g<br>KI-67: 20%<br>TUNEL:56%	human glioma cells<br>mice	[131]
Purchased	RP: (70,90)%<br>RC: (160,170)%<br>RR: (1.5,1.7,2)%<br>LVSP: (75~110)%<br>CF: (3.2~6.5)(ml/min)	MTT	in vitro<br>in vivo	–	SOD: 0.115± 0.025 (U/mg protein)<br>GSH-Px: 0.753 ±0.048 (U/mg protein)	mouse	[90]

		+dP/ + dtmax: (80~110)(mmHg/s) IS: (40~70)%				MDA: 4.38± 0.57 (nM/mg protein)		
	purchased	EDU levels of H4: (50,55)% EDU levels of U251: (28,30)% EDU levels of A172: (35,40)% migration of H4: (38,42,58)% migration of U251: (20,40,58)% migration of A172: (25,38,58)% wound healing H4: (40,59)% wound healing U251: (40,60)% wound healing A172: (42,56)% apoptosis in H4: (17,30)% apoptosis in U251: (21,35)% apoptosis in A172: (19,37)%	MTT	In vitro In vivo	−	colony formation H4: (50,56)% colony formation U251: (30,42)% colony formation A172: (22,50)% tumor weight U251: (1,2.5,2.8)g tumor weight A172: (1.3,2.5,2.6) g TUNEL-positive U251: (20,25,42) % TUNEL-positive A172: (25,40,48)% KI-67 U251: (10,16,41)% KI-67 A172: (10,15,25)% p65 U251: (10,21,41)% p65 A172:(30,59,60)% P53 U251: (20,30,58)% P53 A172: (18,19,28)%	mouse	[115]

Coptis chinensis inflorescence	P38: 1.5%<br>p-P38: 0.5 %<br>ERK: 2%<br>p-ERK: 0.2 %<br>JNK: 1 %<br>p-JNK: 0.8 %<br>Keap1: 1.6 %<br>Nrf2: 1%<br>NQO1:1.4%<br>GST: 0.8 %<br>HO-1: 1.3 %	MTT	In vitro	CE-treated	100μl	mice	[71]
Purchased	IL-2 secretion: (80,81)(U/ml)<br>IL-4: 2 ng/ml<br>IL-5: 3.5 ng/ml<br>IL-10: 5 ng/ml<br>MIP-1α: (13~20)(ng/ml)<br>MIP-1β: (13~21)(ng/ml)<br>RANTES: (2~3)(ng/ml)<br>P24 in Vδ1(-): (8~10)(ng/ml)<br>P24 in Vδ1(+): (4~7)(ng/ml)	_	_	DMSO	CFSE in Vδ1 cells:50%<br>CFSE in Vδ2cell:70%<br>CFSE in αβT cell:25%<br>CD25 in Vδ1:50%<br>CD25 in Vδ2 cell:85%<br>CD25 in αβT cell:20%	cell	[109]
Chrysanthemum morifolium	cell viability: 94.1 ± 5.7%<br>γ-irradiated A549 cells: 80 %	MTT	In vivo	_	_	human	[95]
Cirsium japonicum	90HCJ :(3.02 ± 0.12)(mg/g DW)<br>70HCJ: (3.24 ± 0.06)(mg/g DW)<br>SCJ: (4.11 ± 0.02)(mg/g DW)	MTT	In vivo<br>In vitro	DMSO	_	rat	[83]

		FCJ: (6.96 ± 0.02)(mg/g DW)						
	Jatropha pelargoniifolia	-	MTT	In vitro	Cytotoxicity IC50	25.16 ± 1.5* 13.17 ± 0.9	A549 human lung	[116]
	purchased	bone resorption:(100,60,20)% RANKL:(3.5,3,2.5)pg/ml	MTT	In vivo In vitro	-	-	mouse	[88]
	Mentha haplocalyx	PEG concentration:(98,60,60,70,48)% (NH4)2SO4:80%	ATPF	-	-	-	rat	[84]
**Anti-chlamydial**	*Mentha arvensis*	H.C.V.: 99.0 ± 2.0% (at C: 100 μM) H.C.V.: 97.0 ± 1.3% (at C: 10 μM)	nd	In vitro	-	-	*Chlamydia pneumoniae* cell line (CWL-029)	[47]
**Anticonvulsant**	*Chrysanthemum boreale*	sleeping latency: (2.8,2.9)min total duration: (95,110) min onset time:(80,90)sec T. E: (220,240)sec Mortality:(290,320) sec	HPLC	In vivo	Chloropromazine	Onest time: 43.7 ± 4.81 s T.E :163.7 ± 7.43 s Mortality:200.4 ± 13.9 s	pentobarbital-induced mice	[86]
**Antidepressant**	purchased	N.D. (at D: 14 mg/kg)	Locomotor activity	In vivo	-	-	mice	[85]
	Cirsium japonicum	I.D.: 110 s (at D: 10 mg/kg)	Forced swimming	In vivo	Imipramine	I.D.: 90 s (at 5 mg/kg)	mice	[118]
**Antidiabetic**	Purchased	leukocyte rolling: 15 tes/6mm^2 eukocyte sticking: 8 tes/6mm^2 FCD:41 venular diameter: $75\mu M$	femoral vein	In vivo	_	_	Hamster	[105]

	Chrysanthemum zawadskii	HBA1C: (4.9~5)%<br>AUC glucose: (2800~3000)<br>scored for atrophy: (3~3.5)	-	In vivo	metformin hydrochloride	Insulin: (480~500)(pg/Dl)	mice	[98]
**Antifungal**	*Lippia rubella*	MIC: >125 μg/mL<br>MIC: >125 μg/mL<br>MIC: 125 μg/mL	-	In vitro	Amphotericin B	MIC: 0.250 μg/mL<br>MIC: 0.125 μg/mL<br>MIC: 0.125 μg/mL	*Candida albicans* (ATCC-10231)<br>*C. parasilopsis* (ATCC-22019)<br>*Cryptococcus neoformans* (T1444)	[73]
**Anti-hypersensitivity**	*Chrysanthemum morifolium* Ramat	S.B.P.: 97.5% (at D: 25 mg/kg)<br>D.B.P.: 99% (at D: 25 mg/kg)	-	In vivo	Captopril	-	rat	[132]
	Chrysanthemum indicum	S.B.P.: 170% (at D: 75mg/kg, 1st week)<br>S.B.P.: 175% (at D: 75mg/kg, 2nd week)<br>S.B.P.: 185% (at D: 75mg/kg, 4th week)<br>S.B.P.: 182% (at D: 75mg/kg, 6th week)		In vivo	-	-	rat	[36]

S.B.P.: 175% (at D: 150mg/kg, 1st week)
S.B.P.: 172% (at D: 150mg/kg, 2nd week)
S.B.P.: 180% (at D: 150mg/kg, 4th week)
S.B.P.: 183% (at D: 150mg/kg, 6th week)

D.B.P.: 130% (at D: 75mg/kg, 1st week)
D.B.P.: 132% (at D: 75mg/kg, 2nd week)
D.B.P.: 138% (at D: 75mg/kg, 4th week)
D.B.P.: 140% (at D: 75mg/kg, 6th week)

D.B.P.: 130% (at D: 150mg/kg, 1st week)
D.B.P.: 130% (at D: 150mg/kg, 2nd week)
D.B.P.: 138% (at D: 150mg/kg, 4th week)
D.B.P.: 135% (at D: 150mg/kg, 6th week)

M.A.P.: 144% (at D: 75mg/kg, 1st week)
M.A.P.: 146% (at D: 75mg/kg, 2nd week)

		M.A.P.: 155% (at D: 75mg/kg, 4th week)<br>M.A.P.: 155% (at D: 75mg/kg, 6th week)<br><br>M.A.P.: 147% (at D: 150mg/kg, 1st week)<br>M.A.P.: 145% (at D: 150mg/kg, 2nd week)<br>M.A.P.: 152% (at D: 150mg/kg, 4th week)<br>M.A.P.: 150% (at D: 150mg/kg, 6th week)						
	Buddleia cordata	____	__	In vitro	Acanthamoeba	Acanthamoeba polyphaga :4.0,2.0,0.25,0.03125,0.125 mg/ml<br>Acanthamoeba castellanii: 0.5,4.0,0.25,16.0,0.03125 mg/ml	cell	[57]
**Anti-inflammatory**	purchased	Linarin:0, 2.5 , 5 , 20 , 40 (μg/ml)(ConA :10 μg/ml)<br>Linarin:0, 2.5 , 5 , 20 , 40 (μg/ml)(LPS:50 μg/ml)<br>Linarin: (6.25, 12.5, 25, 50,100 μg/ml)(LPS:10ng/ml)	-	In vivo	LPS-Induced Cytokine RAW 264.7	C.V.M:(ConA)(linarin:2.5 μg/ml) 2.4<br>C.V.M:(LPS)(linarin 0 μg/ml) 0.6<br>N:(LPS)(linarin 0 μg/ml):43 uM	mice	[129]
	Buddleia cordata	Linarin:(2.5 mg/kg) 46.5%	Extract of plant	In vivo	_	IA: 89.0 (73.7—110.0) mg/kg<br>A: 0.6 mg/kg	Mice and rats	[130]

	Linarin: (10 mg/kg) 66.5% Linarin: (10 mg/kg) 82.6%						
purchased	linarin (0, 5, 10, 20, or 30 μM) for 24 h	MTT method	In vitro	RAW264.7 macrophages	-	mouse	[133]
Chrysanthemum zawadskii	TRAP+ MNCs/well : 450 (linarin:0 μM) 320(linarin:1 μM) 180(linarin:3 μM) 120(linarin:10 μM)	EMSA	vitro and in vivo	Collagen-Induced Arthritis	AI:6.1 ± 1.99 at 1 mg/kg, 4.7 ± 1.12 at 10 mg/kg, and 2.8 ± 1.09 at 100 mg/kg(41 days)	mice	[134]
Mentha haplocalyx	IL-1β: (50,45,35,32) (pg/ml) TNF-α: (7000,5800,4000,4500)(pg/ml) IL-6: (14800,14750,12000,10000)(pg/ml) p-IκBα: (0.4,1,0.8,0.7,0.7)(30 min) p-IκBα: (0.3,0.5,0.4,0.2,0.1)(4 h)	_	In vitro	RAW264.7	CV:O(65~110)(%)	Mouse	[76]
Cirsium japonicum	_	MTT	In vivo In vitrio	Dexamethasone (DXM)	NI: (10,15,20)(%)	rat	[17]
Cirsium japonicum	_	_	_	LPS	CV: (83.35 ± 2.22)(%) NI: (10.33 ± 1.61)(%)	Rat mouse	[20]

						IP: (36.19 ± 16.17)(%)		
	purchased	-	lucigenin chemiluminescence	In vivo In vitro	LPS	LI: (4,3.5,2.1,1.5) CD41: (43,30,20,15)(%)	mice	[135]
	Buddlejae Flos	NO: 100~125 μmol/L TNF-*α*: 30~40 pg/mL IL-1β: 20~25 pg/mL	HSCCC	In vivo	HUVEC and LPS	–	human	[136]
	Flos Chrysanthemi Indici	(20,40,80)μ*M* NO concentrations (20,13,10) μ*M* PGE2:(290,250,190)pg/ml	-	In vitro	LPS	-	RAW264.7 macrophage cells	[92]
	purchased	IL-6:(150,130,100,75)pg/ml TNF-*α*:(170,140,100,75)pg/ml PGE2:(800,700,600,400)pg/ml NO:(200,160,140,100)pg/ml Collagen II:(2000,2500,3000,3200)pg/ml Aggrecan:(500,600,650,700)pg/ml MMP13:(250,210,180,130)pg/ml ADAMTS-5:(500,430,380,300)pg/ml		In vibo In vitro	IκB*α* in cytoplasm and p65 in nuclear TLR4	P65:(3,2,1) IκB*α* :(0.25,0.6,1) TLR4:(2.5,1.8,1)	human	[89]
	purchased	cell viability:100% NO:(20,18,15,12)μM	TCMSP	In vitro	-	-	Mouse	[93]

		TNF-α:(380,420)pg/ml						
	Flos Chrysanthemi indica	liver index comparison:(0.055,0.048, 0.052)<br>TC content:15μmole/mg protein<br>Relative FSN mRNA level:(075,0.6,0.5)<br>Relative SCD1 mRNA level:(50,55,78)	-	-	SEM	TG (mmol/L): 1.00 ± 0.29<br>TC (mmol/L) 1.98 ± 0.26<br>HDL-c (mmol/L) 0.96 ± 0.12<br>GLU (mmol/L) 3.83 ± 0.59<br>LDL-c (mmol/L) 0.82 ± 0.14	rat	[97]
**Antipyretic**	*Buddleia cordata*	I: 46.4% (at D: 10 mg/kg)<br>I: 69.0% (at D: 50 mg/kg)<br>I: 86.6% (at D: 100 mg/kg)	yeast-induced hyperthermia test	In vivo	Acetaminophen	I: 68.0% (at D: 200 mg/kg)	rat	[101]
**Anti-Osteoarthritic**	*Chrysanthemum zawadskii* var. *latilobum*	IL-1β:30pg/ml<br>TNF-α:130pg/ml			MMPs<br>Sox9	1,2,1.5<br>1,1.5,1.3	Human	[75]
**Antivirus**	prepared	Lb:50%(at D:40μg/ml)<br>Lb:60%(at D:20 μg/ml)<br>Lb:75%(at D:10 μg/ml)<br>LB: 35%(at D:5 μg/ml)<br>LT:40%(at D:40μg/ml)<br>LT:50 %(at D:20 μg/ml)<br>LT:60%(at D:10 μg/ml)<br>LT: 40%(at D:5 μg/ml)#	MTT method	in vitro	5lg/mL significantly increased T and B lymphocyte	I: 1 μg/ml (5 min)<br>BLIN treating: 0.6 μg/ml<br>I: 0.98 μg/ml (2h)<br>BLIN treating: 1 μg/ml<br>I: 1 μg/ml (10h)<br>BLIN treating: 0.3 μg/ml	duck virus hepatitis (DVH)	[106]
	purchased	C.S: 70 U/mL (IL.2-on clone 1C116 and 2C21)	_	In vitro	Vδ1-TCR or Vδ2-TCR	Vδ1:45%<br>Vδ2:85%<br>αβT:20%	HIV-1 in CD4+ NKT cells	[109]

		C.S: 72 U/mL (IL.2-on clone 1C116)<br>C.S: 18 ng/ml (at linarin: 100 μg/ml) (MIP-1β)<br>C.S: 15 ng/ml (at linarin: 100 μg/ml) (MIP-1α)<br>C.S: 7 ng/ml (at linarin: 10 μg/ml) (IL-13)<br>C.S: 5 ng/ml (at linarin: 100 μg/ml) (IL-5)<br>C.S: 2.8 ng/ml (at linarin: 100 μg/ml)(RANTES)						
	purchased	TNF-α: 40 pg/ml	CDOCKER	In vivo<br>In vitro	OD	_	cell	[110]
**Cell protectivity**	prepared	BLIN:20,10,5,2.5 μg/ml	MTT method	In vitro<br>in vivo	hepatoprotective and antioxidative	I:69.3%<br>Treat:35.5%	duck virus hepatitis (DVH)	[107]
**Estrogenic**	*Cirsium japonicum* var. *maackii*	Linarin:10,30,100 μg/ml	Extract of plant	In vitro	estrogen receptor (ER)	Relative ER avtivity:100 %	human breast cancer (MCF-7)	[19]
**Neuroprotective**	*Valeriana officinalis*		Rotenone method and new genomic method	In vitro	binding SUR1	(TH): -9.3 Kcal/mol<br>SUR:-5.9 Kcal/mol	human PD brain	[82]
**Photo protectivity**	*Buddleja scordioides*	UV_B: 312 nm(0,20,40,40,80 min)	Extract of plant	In vitro	Escherichia coli	Time: 80 min(log 0)	guinea pigs	[137]
	Buddleja scordioides	BCME:5-300 μg/ml<br>UVB 224, 290 and 324: nm	Extract of plant	In vitro<br>In vivo	SKH-1	-	mice	[94]
**Phytotoxicity**	*Zanthoxylum affine*	ATP synthesis:(40~100)%	Holm–Sidak and Dunn's method	In vivo<br>In vitro	DCMU	L. sativa (:0.29 ± 0.03)g (58.4 ± 6.0)%	Lactuca sativa and Lolium perenne	[72]

		Electron transport: (55~100)%				L. perenne: (0.95 ± 0.04)g(69.0 ± 2.9)%		
**Sedative effect**	purchased	V.R.: 75% (at D: 7 mg/kg)	hole board	In vivo	-	-	mice	[85]
	Valeriana officinalis	LN: 4,7,14 mg/kg	Extract of plant	In vivo	-	Sleeping time median, interquartile range: 0 , 1700,900(s)	Swiss mice	[126]
	Valeriana officinalis	LN:2.0,6.0,12,0 mg/kg	_	In vivo	-	Cumulative activity: 1254 ± 130 (s) 1335 ± 134 (s) 1197 ± 151 (s)	mice	[138]
	Chrysanthemum boreale	LN:10,20 mg/kg	-	In vivo	pentobarbital-induced	Total duration:98,103 (min)	mice	[86]
**Sleep enhancing**	purchased	S.T.: 1320 s (at D: 14 mg/kg)	Sodium thiopental-induced	In vivo	-	-	mice	[85]
**Spasmolytic**	*Leonards japonicus*	___	_	In vivo	uterine smooth muscle	contractile activity: (5.7.11)g*n/10 min contractile tension: (0.2,0.5,0.8)(ΔT,g) contractile frequency:	rat	[50]

						(0.6,0.9,1.2)(F, n/10 min)		
**Kidney against Ischemia/Reperfu sion Injury**	purchased	BUN:40<br>SCr:110<br>KIM-1:2.8<br>IL-12<br>p40 mRNA:1.5<br>relative mRNA expression µM,:<br>(20,30,40)µM<br>(3.5,3,2)	CCK8	In vivo<br>In vitro	DMSO	$p < 0.05$	rat	[91]

A: anti-inflammatory activity, ALP: alkaline phosphatase, AUC: area under the curve, AI: arthritis index, A.W.: Average writhing, BCME: *Buddleja cordata* methanolic extract, BHT: butylated hydroxytoluene, BSP: bone sialoprotein, BUN: blood urine nitrogen, C: concentration, ConA: concanavallin A, CP: cell proliferation, CS: cytokine secretion, CV: cell viability, CVM: cell viability max, D: dosage, DBP: diastolic blood pressure, D.L.: detection limit, DRR: Dyskinesia recovery rate, EMSA: electrophoretic mobility shift assay, FBS: fatal bovine serum, FCD: functional capillary density, FCJ: freeze-dried, HCJ: hot-air-dried at 90 °C, HCV: host cell viability, HUVEC: human umbilical vein endothelial cell, I: inhibition, IA: inhibition of abdominal writhing, I.D.: Inhibition dosage, ID: immobile duration, IP: inhibition PGE2, IS: infarct size, LI: lung injury, LN: linarin, LPS: lipopolysaccharide, M: mineralization, MAP: mean arterial pressure, MDA: malondialdehyde; MIC: minimum inhibitory concentration, N: nitrite, nd: not determined, NI: no inhibition, OCN: Osteocalcin, P: protection, P_Bim: Bim phosphorylation, P_STAT3: STAT3 phosphorylation, PCO: protein carbonyl; PD: Parkinson’s disease, RANKL: receptor activator of nuclear factor-kB ligand; RC: relative activity of Caspase-3 on H2C9, RHR: relative histamine release, RP: relative activity of proliferation on H9C2, RR: relative amount of ROS, RUNX2: runt related transcription factor 2, SBP: systolic blood pressure, SCJ: shade-dried, SCr: serum creatinine, ST: sleeping time, TH: tyrosine hydroxylase, V: variation, VR: vehicle response.
